# A New Integrated System for Assistance in Communicating with and Telemonitoring Severely Disabled Patients

**DOI:** 10.3390/s19092026

**Published:** 2019-04-30

**Authors:** Radu Gabriel Bozomitu, Lucian Niţă, Vlad Cehan, Ioana Dana Alexa, Adina Carmen Ilie, Alexandru Păsărică, Cristian Rotariu

**Affiliations:** 1 Faculty of Electronics, Telecommunications and Information Technology, “Gheorghe Asachi” Technical University, Iaşi 700050, Romania; vlcehan@etti.tuiasi.ro (V.C.); alexpasarica@gmail.com (A.P.); 2 Faculty of Electrical Engineering, Energetics and Applied Informatics, “Gheorghe Asachi” Technical University, Iaşi 700050, Romania; luc@rms.ro; 3 Department of Medical Specialities II, Faculty of Medicine, “Grigore T. Popa” University of Medicine and Pharmacy, Iaşi 700115, Romania; ioana.b.alexa@gmail.com (I.D.A.); adinacarmenilie@yahoo.com (A.C.I.); 4 Clinical Hospital “Dr. C.I. Parhon”, Iaşi 700503, Romania; 5 Faculty of Medical Bioengineering, “Grigore T. Popa” University of Medicine and Pharmacy, Iaşi 700115, Romania; cristian.rotariu@umfiasi.ro

**Keywords:** assistive technology, communication system, disabled patients, eye tracking, human–computer interfaces, physiological parameters, telemonitoring, user interface, web browser, wireless sensors

## Abstract

In this paper, we present a new complex electronic system for facilitating communication with severely disabled patients and telemonitoring their physiological parameters. The proposed assistive system includes three subsystems (Patient, Server, and Caretaker) connected to each other via the Internet. The two-way communication function is based on keywords technology using a WEB application implemented at the server level, and the application is accessed remotely from the patient’s laptop/tablet PC. The patient’s needs can be detected by using different switch-type sensors that are adapted to the patient’s physical condition or by using eye-tracking interfaces. The telemonitoring function is based on a wearable wireless sensor network, organized around the Internet of Things concept, and the sensors acquire different physiological parameters of the patients according to their needs. The mobile Caretaker device is represented by a Smartphone, which uses an Android application for communicating with patients and performing real-time monitoring of their physiological parameters. The prototype of the proposed assistive system was tested in “Dr. C.I. Parhon” Clinical Hospital of Iaşi, Romania, on hospitalized patients from the Clinic of Geriatrics and Gerontology. The system contributes to an increase in the level of care and treatment for disabled patients, and this ultimately lowers costs in the healthcare system.

## 1. Introduction

In recent years, national and international efforts have been made to increase the quality of care for people with various disabilities while maintaining costs that are supportable by society. To achieve these conflicting requirements, researchers have made efforts to introduce various equipment and techniques that involve progressively more sophisticated components of informatics and telecommunications.

When it comes to caring for severely disabled people, the requirements for round-the-clock surveillance are universally accepted. As a rule, several trained people, who are responsible for medical compliance and surveillance, attend to the physiological needs of disabled patients. However, the former can do very little for the psychological needs and the quality of life of the latter.

Assistive technology ensures greater independence for people with disabilities, allowing them to perform tasks that are otherwise impossible or very difficult to accomplish. The proposed system provides these possibilities by improving or changing the way that patients interact with the objects and equipment necessary to perform that task [1,2].

Progress in the field of electronics in the last few years has generated a real interest in the development of new assistive systems adapted for different types of patients. These systems are very useful for medical investigations and observation and also contribute to an increase in the patient’s quality of life.

Two research directions have been developed for the care of patients with severe disabilities: (1) telemonitoring physiological parameters; and (2) ensuring communication with disabled patients. Both directions, alongside remote diagnostics, GPS tracking, and others, are included in what can be called telemedicine.

Nowadays, telemonitoring is considered an excellent method for diagnosis and surveillance, as proven by numerous studies and projects that are finalized or still in progress [3,4,5,6,7,8]. Some of the most significant projects at the international level include Universal Remote Signal Acquisition for Health (U-R-SAFE) [9] and CodeBlue [10], which can be considered as reference projects carried out at Harvard University. Many systems have been implemented to assist people with cardiac diseases, such as, Health Early Alarm Recognition and Telemonitoring System (HEARTS) [11]; Enhanced Personal, Intelligent and Mobile System for Early Detection and Interpretation of Cardiological Syndromes (EPI-MEDICS) [12]; and the real-time health monitoring system for remote cardiac patients using a Smartphone and wearable sensors, as described in Reference [13].

The advanced technologies applied by many researchers in different technical fields meet the needs of patients with autism [14] and dementia. Moreover, human–robot interaction systems [15] are now able to recognize gestures that are usually employed in human non-verbal communication [16] to aid visually impaired people in performing ordinary activities [17,18] or capturing non-visual communication [19]. These systems also facilitate the rehabilitation and augmentation of children who suffer from motor and social function impairments [20] and people who have suffered a stroke [21,22]. Lately, particular attention has been paid to the implementation of assistive systems for neuromotor rehabilitation to enhance the recovery and societal reintegration of severely disabled patients [23].

Despite all efforts, telemedicine has not yet become a standard procedure in current medical practice, with many aspects still under study. Some relevant considerations are presented in Reference [24]. One of the reasons is the distinctiveness of the systems used for telemonitoring (like the systems described in References [25,26]) and telecommunication with disabled patients (like the systems described in References [27,28]). These systems are usually realized by different researchers or providers [29,30,31,32] and are not compatible. The direct consequences are difficulties acquiring, installing, and maintaining these systems and—last but not least—training staff and patients. Moreover, these systems have high acquisition costs.

In general, the assistive systems presented in the literature have limited functions and address a specific type of diseases (e.g., cardiovascular diseases, neuromotor rehabilitation after a stroke). Our purpose is to implement a new complex system that includes two functions: (1) communication with disabled patients by using keywords technology and (2) telemonitoring their physiological parameters. Unlike other systems, the two functions of the proposed system are software-controlled using a new WEB application. One of the advantages of the proposed system is that it is easy to configure and can be adapted to patients’ needs. These needs can be detected by using switch-type sensors that are suitable for different degrees of disability or by using an eye-tracking interface for communication with severely disabled patients.

The proposed system represents the results of many years of the authors’ work performed as a part of several research contracts won by competition [33,34,35]. Partial results obtained during these research activities have been presented in several papers published over the years [36,37,38,39,40], but this paper is the first comprehensive presentation of the whole system.

The main beneficiaries of assistive technologies are patients with severe disabilities, which range from severe neuromotor sequelae to permanent bed immobilization due to various and multiple causes: advanced chronic diseases (cardiovascular, renal, respiratory), osteoarticular pathologies with functional impotence, neglect, depression, etc. These patients usually have a low quality of life because they lack the ability to communicate with the people surrounding them. Connecting with other people, even at a basic level, may ameliorate physical and psychological needs, thus improving their life quality and self-esteem.

The paper is organized as follows. Section 2 describes the hardware and the software of the proposed system, as well as its functions and operating modes. System testing done in laboratory conditions and performed on hospitalized patients are illustrated in Section 3. Section 4 presents the main results and discussions. In Section 5, some conclusions are drawn.

## 2. Materials and Methods

The system illustrated in this paper, implemented at the prototype level, is the result of an interdisciplinary collaboration of specialists from the fields of electronics, telecommunication, computer science, and medicine. The proposed system helps the healthcare system move toward a drastic decrease in care costs. It functions by using keywords technology for two-way communication with severely disabled patients and uses the Internet of Things (IoT) concept to implement the wireless and wearable sensor network, which is used for telemonitoring the physiological parameters of the patients. Both functions of the system (communication and telemonitoring) share the same hardware platform (except for the sensors used for capturing the physiologic parameters) and most of the software components.

From the communication perspective, the target consists of patients that are able to hear and/or see and understand but are unable to easily communicate (because of a neuromotor handicap, severe dyspnea, depression) using conventional methods, such as speech, writing, or hand gestures. Usually, these patients are able to make only limited movements, such as muscle contractions (raising a forearm, moving a finger or foot) or, as is the case in most situations, eye movements and blinking. Whatever the situation, the problems always remain the same: Patient ↔ Caretaker communication and medical investigation and observation.

The purpose of telemonitoring physiological parameters is to efficiently assess the patient’s current condition, allowing rapid intervention if necessary. The system constantly observes the previously programmed physiological parameters and sends alarms to the caretaker when the values of the monitored parameters are beyond normal limits. All monitored parameters, alarms, and caretaker interventions are recorded in the Server memory for later analysis.

### 2.1. Description of Assistive System Hardware

**Figure 1:**
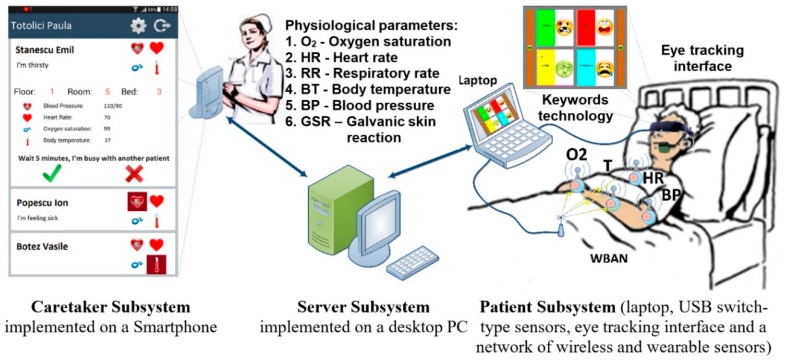
Structure of the proposed integrated system for assistance in communicating with and telemonitoring severely disabled patients.

The proposed assistive system is designed for severely disabled patients to enable the simultaneous two-way communication and telemonitoring of the patient’s vital physiologic parameters. The structure of the system is illustrated in Figure 1; it includes the following three subsystems: Patient, Server, and Caretaker. The Server Subsystem is represented by a conventional desktop PC, and the Caretaker Subsystem consists of a Smartphone device (Figure 1).

The Patient Subsystem includes a communication module implemented by different types of human–computer interfaces and is used for communication through keywords technology, a telemonitoring module, which is represented by a network of wireless and wearable sensors that capture physiological parameters, and a laptop (tablet PC or other devices) for the collection of communication and monitored data values.

#### 2.1.1. Communication Module

**Figure 2:**
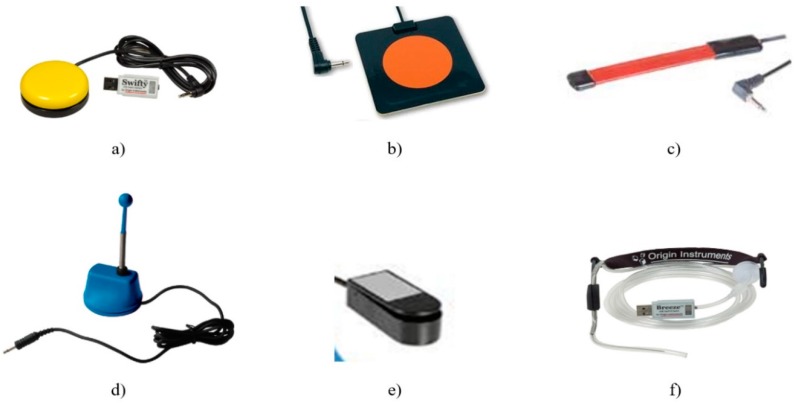
Different types of USB switch-type sensors used for keywords-based detection: (**a**) hand switch-click (mouse button switch) with a Swifty USB switch interface; (**b**) pal pad flat switch; (**c**) ribbon switch; (**d**) wobble switch; (**e**) foot switch; (**f**) sip/puff breeze switch with headset.

**Figure 3:**
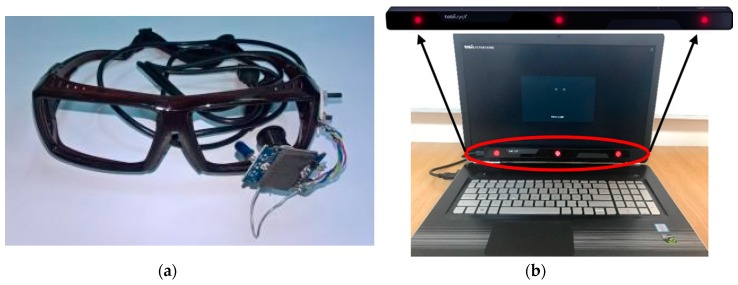
Eye-tracking-based interfaces: (**a**) head-mounted eye-tracking interface consisting of an infrared video camera mounted on an eyeglasses frame; (**b**) remote eye-tracking interface consisting of a commercially available infrared sensor mounted on the patient’s laptop.

The communication module of the proposed assistive system is based on human–computer interfaces that can be adapted for different types and degrees of disability. These devices are used for the patient’s needs detection and are implemented in our system depending on the user’s physical condition. The devices that can be used with this module are the following:

- Switch-type sensors are for patients who can perform controlled muscular contraction. These sensors are USB-connected to the laptop and adapted to the physical condition of the patient. Some examples of the switched-based sensors used by this system are illustrated in Figure 2: hand-controlled sensors, including a hand switch-click (Figure 2a), pal pad flat switch (Figure 2b), ribbon switch (Figure 2c), and wobble switch (Figure 2d); foot-controlled sensors, such as a foot switch (Figure 2e); sip/puff breeze switch with headset, as illustrated in Figure 2f (the leading sip-puff system for individuals with motor disabilities and limited dexterity).
- Eye-tracking-based interface is for fully immobilized patients who cannot perform any controlled movement, except eyeball movement, and who are unable to communicate verbally (Figure 3).

Eye tracking is the process of measuring either the point of gaze or the motion of an eye relative to the head. An eye tracker is a device for measuring eye positions and eye movement [41,42,43,44]. Lately, gaze direction detection techniques have developed in two basic directions: electro-oculography (EOG), which was used by our team in the ASISTSYS project [34], and digital image processing using video-oculography (VOG), which is used in the present system.

Digital image processing techniques use video cameras in the visible and infrared (IR) spectrum to take video eye images, which are subsequently processed frame by frame using special software installed on a computer to extract the coordinates of the eye pupil. Currently, increasing computing power has led to the diversification of pupil detection algorithms (PDAs). The advantages of the methods based on the analysis of the eye images provided by video cameras lie in their versatility; moreover, they are practically independent of the individual characteristics of the patient’s eye.

The proposed assistive system uses two types of real-time eye-tracking interfaces to communicate with severely disabled patients:

- head-mounted device (Figure 3a), which measures the angular position of the eye with the head as the point of reference [45];
- remote device (Figure 3b), which measures the position of the eye with the surrounding environment as the point of reference and is implemented with a commercially available interface developed by Tobii [32]; the IR sensors are placed at the base of the screen.

The head-mounted eye-tracking interface illustrated in Figure 3a consists of an infrared video camera mounted on an eyeglasses frame placed close to the eyes and connected to a Patient Subsystem (laptop) for eye image acquisition and processing.

This communication module is used by patients with severe disabilities who cannot communicate with other people through classical methods: speech, writing, or hand gestures.

The remote device presented in Figure 3b is used by patients who, as a result of their physical condition, cannot support a helmet on their head.

Because numerous types of available sensors can be used for detecting the patient’s needs, our system can be easily adapted and configured to different types of diseases and patients’ needs.

**Figure 4:**
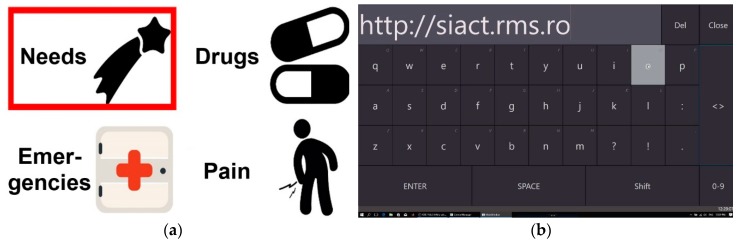
Two-way communication function of the proposed assistive system: (**a**) ideograms/keywords displayed on the patient display; (**b**) virtual keyboard used to build sentences.

The communication function is two-way: Patient ↔ Server ↔ Caretaker. Patient ↔ Caretaker communication relies on keywords technology: the system ensures the successive visualization/audio playback of keywords/ideograms/alphanumeric characters. The patient has the possibility of choosing the desired keyword, as in Figure 4a, or the character needed by using a virtual keyboard, as in Figure 4b. The patient expresses their will or need by selecting a keyword by a technique imposed by the disability: (1) using a switch-type sensor mounted onto the patient’s forearm/finger/leg/sip/puff or (2) by detecting the patient’s gaze direction using the eye-tracking-based interface and performing a video analysis of the eyeball movement. The selected keyword is then sent to the caretaker via the WI-FI network.

The system thus allows patients to use computers to communicate with other people not only for medical investigation but also for other more complex activities. The caretaker’s response is sent to the patient as sound and/or visual by text/ideogram. Consequently, a dialogue may take place between the patient and the caretaker (doctor, nurse, etc.). This is essential for diagnosing patients who are unable to communicate otherwise; using our system, they can answer questions (e.g., “Does it hurt?”, “Yes”; “The leg?”, “No”; “The arm?”, “No”; “The throat?”, “Yes”). Using the keywords technology based on the switch-type sensor, the patients can select only one object (ideogram/keyword) for each phase (Figure 4a). In such cases, the use of alphanumeric characters to build words and phrases would be too slow.

The technique of patient gaze detection by video-oculography (eye pupil image analysis) makes it possible to identify and communicate ideas and complex moods by selecting alphanumerical characters displayed on a virtual keyboard (Figure 4b). This technique involves the display of several characters on the screen simultaneously and the gradual construction of words or phrases using the characters displayed. It can also be used to command the computer to use the Internet and even e-mail. Furthermore, it can be used for reading books, listening to music, and watching TV programs and movies. Of course, all of these options are available to patients who are cognitively able but cannot communicate normally for a limited period of time, such as after surgery. However, these alternatives can be adapted to the patient’s level of cognition, preoccupations, and interests, as they can greatly contribute to increasing the individual’s quality of life.

#### 2.1.2. Telemonitoring Module

The telemonitoring module is based on a wireless and wearable sensor network in which each node is equipped with a sensor for capturing the physiological parameters of patients according to their needs. Thus, each Sensor Node (SN), which represents a medical device in the network, is wirelessly connected to the Central Node (CN), which is USB connected to the patient’s computer, which is routed to the Server Subsystem. The structure of this function is based on the IoT concept: by using sensors, the entire physical infrastructure of the system is interconnected to transmit useful measurement information via the distributed sensor network.

The network management is efficiently controlled by the Server, which transmits the measured data values to the caretaker’s mobile device.

**Figure 5:**
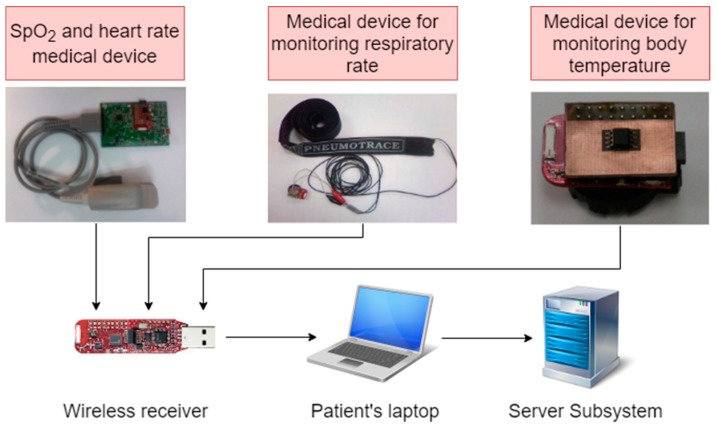
Wireless body area network (WBAN) of telemonitoring module used for the acquisition of the different physiological parameters of patients: SpO_2_, HR, RR, and BT.

The telemonitoring module illustrated in the diagram from Figure 5 consists of a wireless body area network (WBAN) of medical devices attached to the patient’s body for acquiring the following physiological parameters: blood oxygen saturation (SpO_2_), heart rate (HR), respiratory rate (RR), and body temperature (BT). The number and type of monitored parameters can be adapted to the patient’s medical needs and can be selected according to medical recommendations.

The medical devices of the network nodes consist of commercial modules with low energy consumption that capture the above-mentioned physiological parameters.

They are directly connected to the wireless data transmission module (eZ430-RF2500) [46] placed in the network central node, which is equipped with a microcontroller (MSP430F2274) [47] and a radio transceiver (CC2500) [48], and are battery powered.

For measurements of SpO_2_ and HR, a commercially available medical module AFE44x0SPO2EVM [49] is used. The module that uses the AFE4400 circuit is intended for the evaluation of the acquisition and processing of photoplethysmography signals. The measurement of the respiratory rate makes use of a specialized piezoelectric transducer, PNEUMOTRACE [50], mounted around the patient’s thorax. It provides an electrical signal in response to a modification in thoracic circumference due to patient respiration. Body temperature measurements are taken using an integrated temperature sensor TMP275 [51] that is directly connected to the eZ430-RF2500 data transmission module through an I2C serial interface.

The patient’s computer runs a background software application (presented in Section 2.2) that takes the data collected by medical devices and sends them to the Server device for processing; from here, the data are wirelessly transmitted to the caretaker device, where they are displayed in real-time. When it detects that certain limits are exceeded to a level that is considered dangerous, the Server, by automatic data analysis, alerts the caretakers, and their interventions are saved in the Server memory for later analysis. The monitored data values can be processed at any time in order to see the evolution of the patient’s physiological parameters over time. This type of analysis is vital for establishing optimal treatment.

### 2.2. Description of Assistive System Software

The three components of the assistive system (Patient, Server, Caretaker) are connected via the Internet. The operating modes of the system components are controlled by software. In the following, all the system software components are described in detail.

#### 2.2.1. Software Component of *Patient Subsystem*

The software component of the Patient Subsystem was developed for both communication and telemonitoring functions.

The software component of the Patient Subsystem includes the Patient WEB Application (PWA), which is implemented on the Server Subsystem and can be accessed remotely through WI-FI Internet connection by each patient’s device. This network organization of patients’ devices simplifies the software component because the Server manages all the devices through the same configurable and adaptable application for each type of patient.

The software component of the communication function is based on keywords technology, implemented using the Patient WEB Application, which enables both switch-based and eye-tracking-based operating modes for needs detection. Independent of the PWA on the patient’s device is software developed for eye-tracking interfaces. All of these software components are based on algorithms proposed by the authors.

The software component of the telemonitoring function is also included in the Patient WEB Application. It ensures the collection of data provided by the wireless and wearable sensors and the real-time transmission of the monitored values to the supervisor device via the *Server*. The telemonitoring function includes a graphical interface that runs on the patient’s device and is used for the real-time numeric and graphical display of the monitored physiological parameters, alerts resulting from their processing, and the status of each node in the network. The communication protocols used for transmitting monitored data to the Server are also included.

1. Software component of communication function: Operating modes

**Figure 6:**
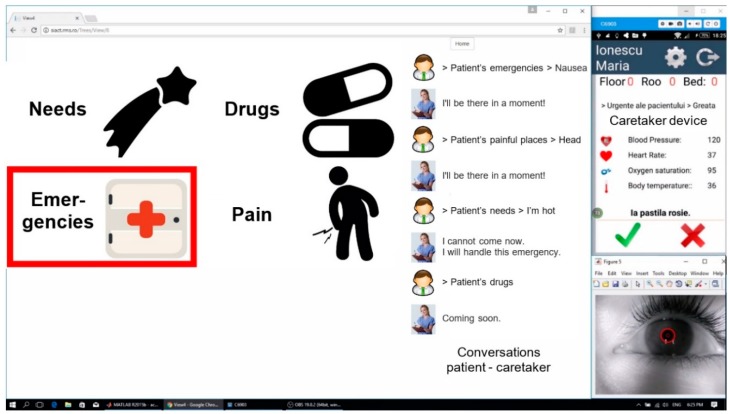
Patient WEB Application for communication using switch-based or eye-tracking-based patient’s needs detection. (Patient device screen: Laptop; Caretaker device screen: Smartphone; raw eye image provided by the IR video camera).

In the case of switch-based communication, PWA runs the ideograms on the user screen by cyclical highlighting (Figure 6). The patient can select an ideogram by activating the switch sensor only while it is highlighted, which lasts for a time interval that is set according to the patient’s experience in using the system. The database with ideograms/keywords is organized hierarchically. Each level from the database contains a set of ideograms/keywords referring to a class of objects defined in ascending order. When the patient selects an ideogram using the switch (which can be of any type, as illustrated in Figure 2), the request is forwarded to the caretaker device via the Server.

**Figure 7:**
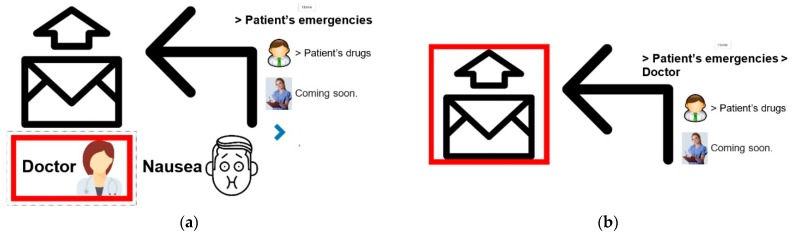
The process of sending a message to the caretaker: (**a**) ideograms belonging to the second hierarchical order after the first selection by the patient; (**b**) ideogram corresponding to the transmission of the wanted message (“Doctor”).

Figure 7 presents the process of sending a message using PWA when the ideogram “Emergencies”, highlighted in Figure 6, is selected. Figure 7a presents the set of ideograms (“Doctor” and “Nausea”), which belong to the previously selected class, and Figure 7b illustrates the last hierarchical level of the three, corresponding to the transmission of the message “Doctor”.

When the patient considers that the message can be sent, s/he selects the “Send” ideogram (Figure 7b) by using one of the previously described techniques, and the message is sent to the app on the mobile Smartphone of the caretaker (nurse, medical staff).

The application running on the patient’s laptop displays the messages sent by the patient during the dialogue with the caretaker on the right-hand side of the screen (Figure 6 and Figure 7). The history of these conversations is stored in the Server memory and can be checked if needed.

It is worth noting that the proposed system has the ability to customize databases with ideograms and keywords organized in the form of trees for each individual patient. Thus, each patient has an account in the PWA that contains a personalized set of ideograms/keywords. The telemonitored physiological parameters for each patient, according to medical recommendations, are also specified in the PWA. Depending on the evolution of the patient’s condition during treatment, this database can be easily updated as needed.

In the case of eye-tracking-based communication, the user controls the PWA by using one of the two types of eye-tracking interfaces illustrated in Figure 3. The patient’s screen is the same as in the previous case (Figure 6), but the ideograms are not cyclically highlighted, as the user is able to fully control the application by using gaze direction.

The operation of the eye-tracking interface is based on the real-time detection of the pupil’s center coordinates in the raw eye image provided by the infrared video camera (as shown in Figure 6). In order to do this, the authors developed an eye-tracking algorithm, which runs independently of the PWA on the patient’s laptop. The purpose of this algorithm is to move the cursor on the screen according to the user’s gaze direction and to perform ideograms/keywords selection by simulating a mouse click. The eye-tracking algorithm includes the following main procedures, which are described in Reference [36] in detail: (1) real-time eye image acquisition; (2) system calibration; (3) real-time pupil center detection; (4) mapping between raw eye image and scene image; (5) ideograms and/or objects selection; and (6) algorithm optimization, which is needed to stabilize the cursor position on the user screen by real-time filtering and high frequency spike canceling from the PDA signals.

In order to perform pupil detection, the dark-pupil technique was implemented [52,53]. Dark-pupil techniques illuminate the eye with an off-axis infrared source such that the pupil is the darkest region in the image (Figure 6) and can be easily detected by using a threshold-based binarization technique, illustrated in Reference [37]. On the other hand, by using this illumination type, a consistent and uniform illumination of the eye can be obtained without any user discomfort.

**Figure 8:**
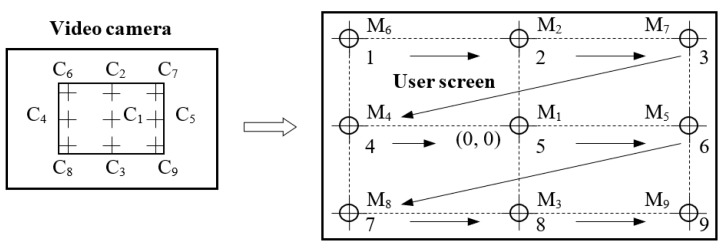
Calibration of the eye-tracking device.

In order to perform eye-tracking system calibration, nine calibration points are displayed on the user screen in the order illustrated in Figure 8 [36]. These points, denoted by M*_i_* (*i* = 1, …,9), represent the calibration points on the coordinate system of the user display. During the calibration process, the user’s gaze (the eyeball) follows these points in the order indicated in Figure 8, with equal pauses between them. Corresponding to these points are another nine resulting points, denoted by C*_i_* (*i* = 1, …,9), in the coordinate system of the eye image (with 640 × 480 resolution) provided by the IR video camera.

The conversion between the eye image coordinates (pupil’s center position) and the user screen coordinates (user’s cursor position) is performed by a special mapping function [54]. The coefficients of the mapping function are obtained during the calibration process when a set of targets in known positions are displayed to the subject and the eye tracker position data are recorded.

The calibration process is performed for each subject who uses the system. The accuracy of the eye tracker operation depends on the successful completion of the calibration stage.

Patients with different ocular diseases (e.g., strabismus) or neurologic diseases who were unable to complete the calibration stage successfully were excluded from these tests and included in the category of patients who were not able to perform their tasks by using the eye-tracking interface.

The pupil center can be detected by using different principles that are illustrated in the literature: the least-squares fitting of ellipse algorithm (LSFE) [55,56], the RANdom SAmple Consensus (RANSAC) paradigm [57,58], circular/elliptical Hough transform-based approaches (CHT/EHT) [59,60], and projection method (PROJ) algorithms [61,62]. On the basis of these principles, we developed original PDAs that are adapted to our eye-tracking interface; the algorithms were tested and compared with the open-source Starburst algorithm illustrated in Reference [63]. Some results on the performance of these algorithms come from the authors’ previous studies, including the algorithms LSFE [64], RANSAC [65], and CHT [36]. Details of the test modes and the results obtained are given in Section 3.1.1.

After detection, the coordinates of the pupil center undergo real-time transformation into cursor coordinates on the user screen by using the mapping function. The pupil detection algorithm delivers two real-time signals corresponding to the cursor position on the user screen in both directions of the coordinate system. In this way, the cursor movement on the user screen is controlled by the user’s gaze direction. In order to perform this action, both types of eye-tracking interfaces illustrated in Figure 3 are used in our system, depending on the patient’s physical condition. In recent years, remote video-based eye trackers, which are preferred by patients, have become increasingly more widespread.

Regardless of the type of PDA used, the user must be able to perform two actions:

- cursor movement on the user screen according to the user’s gaze direction only for visual inspection of PWA objects and
- ideogram/keyword selection by simulating a mouse click when the cursor controlled by the user’s gaze direction is placed in the selection area of the wanted object on the screen.

In order to find the best solution for our system, different ideogram selection methods were investigated. The first method tested relied on the algorithm’s ability to identify voluntary blinking by using the total number of consecutive frames that capture the blinking or using a dynamic threshold for selection [66]. The application requires the user’s attention and concentration, and using the system for a long time can become tiresome. Consequently, this may cause errors in distinguishing between voluntary and involuntary blinking.

The proposed system uses the solution that was evaluated as the best for implementing a mouse click: the user’s gaze direction is focused on the selection area of the wanted ideogram/keyword, and the cursor position is maintained in that area for a certain dwell time. However, this technique can lead to the false selection of ideograms as a result of the Midas touch problem, which involves a random selection of unwanted ideograms followed by the user’s gaze direction [44]. For the optimal operation, the system must be able to differentiate viewing from gaze control. This can be achieved by setting the dwell time according to the user’s ability to use the system.

In order to improve the user’s experience of operating the system, the application provides feedback to the user by highlighting the selection area of the ideogram after its successful selection on the user screen.

Any eye-tracking system is affected by many sources of noise that significantly influence the pupil detection process. These sources cause noise signals that overlap the target signals provided by the eye-tracking algorithm. These include a high-frequency noise component produced by the video camera’s electronic circuits, different errors of the PDA during the detection process, artifacts due to variable and non-uniform lighting conditions, and a low-frequency drift component, which is due to the difficulty of keeping the eye absolutely fixed on a point on the screen [67].

In addition, there are many other sources of noise that should be considered when the eye-tracking system is designed, such as corneal reflection due to the infrared illumination of the eye, physiological tremor of the eye [68], non-constant eye movement made up of saccades (rapid movements) and fixations (short stops) [67], modification of the pupil image shape and position during fixations, obstruction of the pupil by the eyelids or other artifacts, involuntary blinking, and head movements, which produce high frequency spikes in the signals provided by the PDA.

These multiple sources of noise cause the main drawback of this technique, which consists of the difficulty in maintaining the cursor in a fixed position on a selection area of the display for a certain dwell time to perform a selection. In order to increase the cursor stability on the user screen, the eye-tracking algorithm implemented in this system was optimized by introducing different techniques that are based on real-time filtering and high frequency spike canceling from the signals provided by the PDA. The best results were obtained by using the snap-to-point technique, especially for the situation in which the selection area of an ideogram on the user screen is small in size, as is the case in the communication procedure based on a virtual keyboard controlled by eye tracking. By using this technique, it was possible to introduce more complex applications, such as browsing the Internet and accessing personal e-mail, into our communication module.

2. Software component of telemonitoring function

As a network protocol, we decided to use *SimpliciTI* [69] from Texas Instruments to transfer data through the WBAN. *SimpliciTI*’s main features include low memory needs, advanced network control, and sleeping mode support. It is intended to support the development of wireless networks that contain battery-operated nodes and require low data rates.

**Figure 9:**
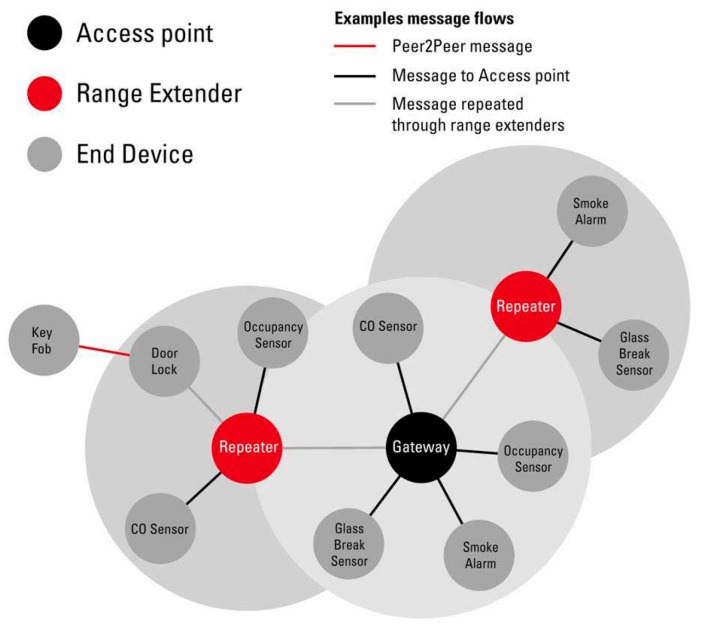
*SimpliciTI* wireless protocol [69].

The eZ430-RF2500 modules connected to medical devices were configured as End Devices (EDs). The same module is connected to the patient’s computer as an Access Point (AP), and several others can be configured as Range Extenders (RE), according to *SimpliciTI* wireless protocol in Figure 9.

**Figure 10:**
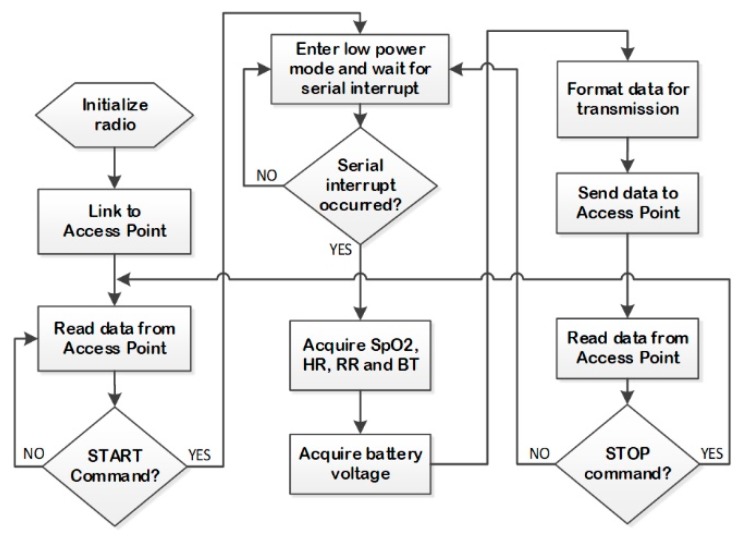
Flowchart of firmware running on MSP430F2274.

The flowchart of the firmware running on the MSP430F2274 microcontroller from the ED is represented in Figure 10. In this instance, the eZ430-RF2500 module is initialized onto the network; then, after a START command, it wakes up to read the SpO_2_, HR, RR, and BT values from the medical devices. Also, MSP430F2274 reads the battery voltage and communicates the data to the central monitoring station through the RE and AP. In order to minimize energy waste, since an important power consumer element is the radio transceiver, the CC2500 enters a low-power mode after each transmission cycle.

**Figure 11:**
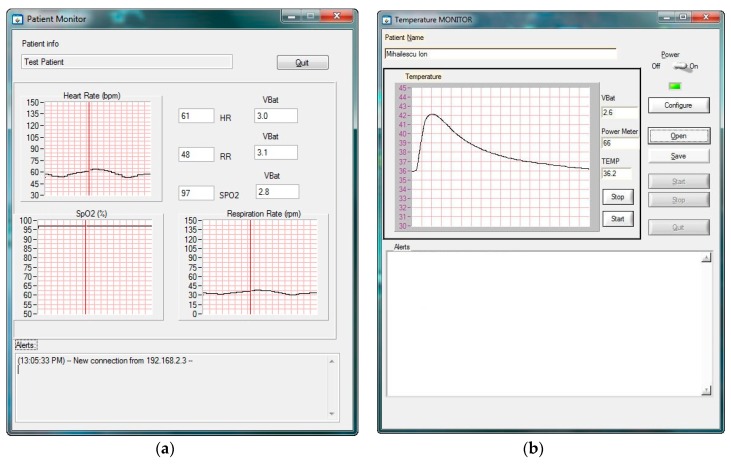
Graphical interface running on the patient’s device used for telemonitoring: (**a**) heart rate, blood oxygen saturation (SpO_2_), and respiration rate [38]; (**b**) body temperature.

A user-friendly Graphical User Interface (GUI) was developed for the patient’s monitor application to display the received measurements and alarms. The GUI running on the central monitoring station was developed using LabWindows/CVI and is shown in Figure 11a [38] and Figure 11 b.

The GUI displays the temporal waveform of the SpO_2_, HR, and RR parameters for the selected patient, together with the status of the node (the battery voltage and distance from the nearby RE or AP). The distance is represented in percent and computed on the basis of received signal strength indication (RSSI) measured from the power of the received radio signal.

In order to alert the caretaker when the normal values of the telemonitored patient’s physiological parameters are exceeded, the proposed assistive system uses an alert detection algorithm, which is implemented in the Server Subsystem.

The physiological conditions that may cause alerts are low SpO_2_ if SpO_2_ < 93%, bradycardia if HR < 40 bpm, tachycardia if HR > 150 bpm, HR arrhythmia if ΔHR/HR > 20% over the last 5 min, HR variability if max HR variability > 10% /the last 4 readings, low body temperature if BT < 35 °C, high body temperature if BT > 38 °C, low RR if RR < 5 rpm, low battery voltage if VBAT < 1.9 V, and a low value for RSSI if the measured RSSI < 30%.

#### 2.2.2. Software Component of *Server Subsystem*

The Server Subsystem is a conventional desktop PC operating as a dispatcher with many functions: it manages the databases of the patients (keywords database, personal data and medical records of the patients, data values of the monitored parameters); it receives, organizes, and processes the data values of the telemonitored physiological parameters of the patients and sends this information to the caretaker devices (Smartphone); it records and organizes the evidence and the history of the patients’ conversations with the caretakers; and it detects alarming situations and alerts whoever may be concerned, among other functions. All of these features are available by running the Patient WEB Application, implemented at the Server level.

Sensitive data are encrypted by the Server Subsystem before being sent to the system clients, so the communication channel is protected regardless of whether the *http* or *https* protocol is used. Medical data are completely anonymized and encapsulated into customized system objects so that nobody can access the medical data of a given patient. Even if the communication system is hacked, the data are unreadable and cannot be linked to any given patient because the patient’s name is never sent out by the Server, as all medical data communicated in the system are assigned to a Globally Unique Identifier (GUID). Only the system members with a specific account and password can access the data. Each user has specific rights and can access only the data for which s/he has received rights from the system administrator.

Since the system uses an Internet connection, the number of patient and caretaker devices that can be connected in the network is unlimited, and these devices can be placed at any distance (in different hospital rooms). All of the system components are controlled by the Server through the Internet network. As a consequence, the system operation (installation, configuration, and administration) is simplified. Since the Patient Subsystem must be periodically adapted and configured to the patient’s needs, the remote administration of these settings represents an important advantage.

**Figure 12:**
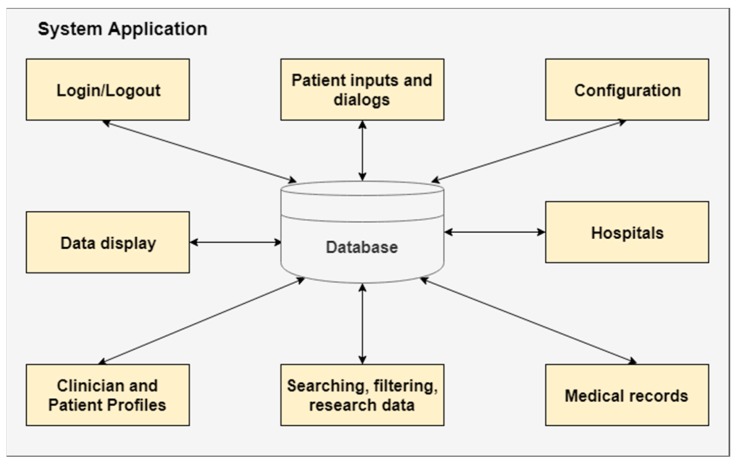
Overview of database responsibilities.

**Figure 13:**
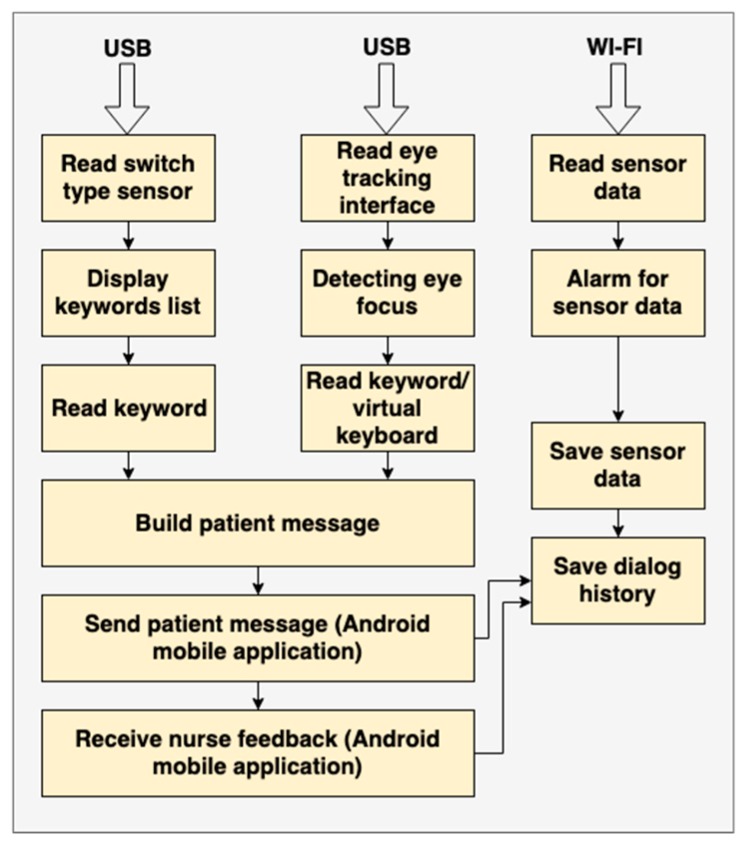
Overview of tasks for each device.

The Patient WEB Application deals with both patient interaction and medical data management (Figure 12). The “Patient inputs and dialogs” block is detailed in Figure 13, where the tasks of each input device are illustrated.

The system uses the new *SignalR* technology [70], which makes the dialogue between the browser and Server very easy to implement [39].

The PWA has many benefits: it does not require installation on the patient’s device, it does not require synchronizations, and it is easy to maintain.

**Figure 14:**
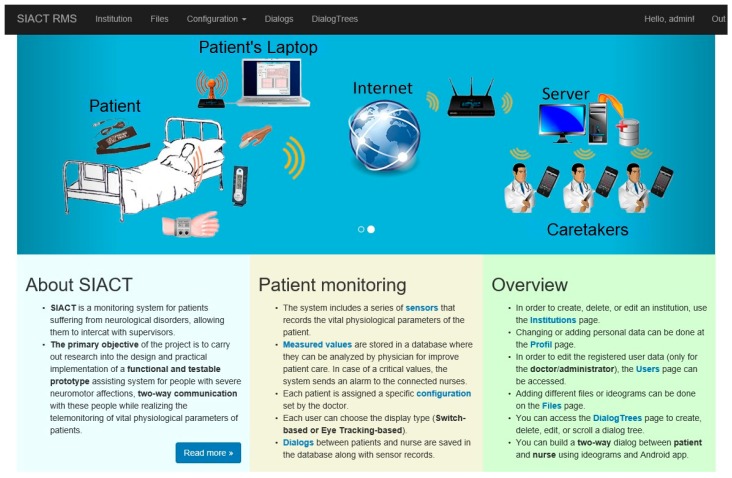
Homepage of the Patient WEB Application.

The homepage of the Patient WEB Application is shown in Figure 14. The PWA can be accessed at http://siact.rms.ro. By accessing this link, PWA can be customized and run by each patient in the network according to their needs and medical recommendations.

In the following, the structure and main functions of this WEB application are presented in detail. The first task of the application is to define the organizational framework in which the assistive system can be used.

The Patient WEB Application is designed to be flexible and easily adaptable to the specificities of each type of patient, depending on the type and degree of disability, and uses switch-based or eye-tracking-based communication modes, as presented previously. For each patient, the personal and medical data are loaded, and the structure and content of the keyword/ideogram database used for communication are built. The PWA sustains the dialogue between the patient and caretaker by building the patient’s message and the conversation page. At the same time, the physiological parameters of the patients are monitored in real-time. When the normal values of these parameters are exceeded, the application will alert the supervisor by means of a message accompanied by a beep that is received on the supervisor’s device, represented here by a Smartphone.

The system administrator assigns one of the following roles to users: admin, doctor, patient, or nurse. Depending on the assigned role, each user has specific access rights.

The communication function of the PWA is based on keywords technology, as previously described. Ideograms accompanied by keywords are intuitive images that characterize a particular state of the patient. The patient can build simple sentences to express a certain need or state by putting the ideograms in a logical sequence. This action is shown in Figure 6 and Figure 7.

The database of ideograms and/or keywords is organized hierarchically as logical trees. They can be customized for each patient. There is a root ideogram to which child ideograms are attached, and child ideograms may expand the idea started from the root. Additional levels of ideograms lead to an accurate description of the patient’s state or need.

**Figure 15:**
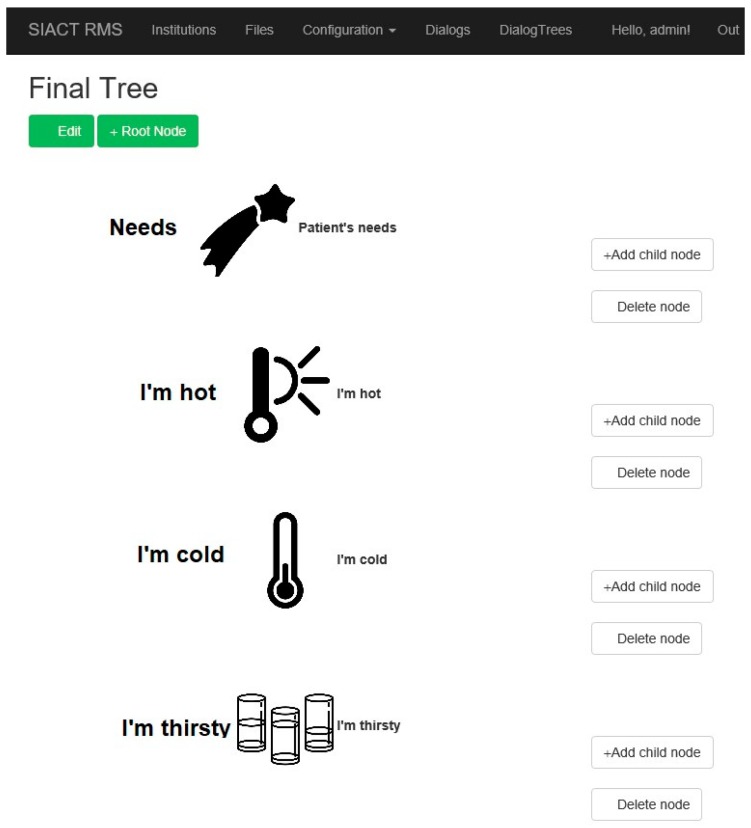
Building of a tree with several hierarchical levels in the Patient WEB Application.

The ideograms used by the system must be personalized for each patient because patients have different pathologies and needs. Each patient has a personal account in the application. Thus, the application has the ability to introduce or remove ideograms from the system database. An example of such a logical tree is illustrated in Figure 15.

Another function of the PWA consists of telemonitoring the patient’s physiological parameters.

**Figure 16:**
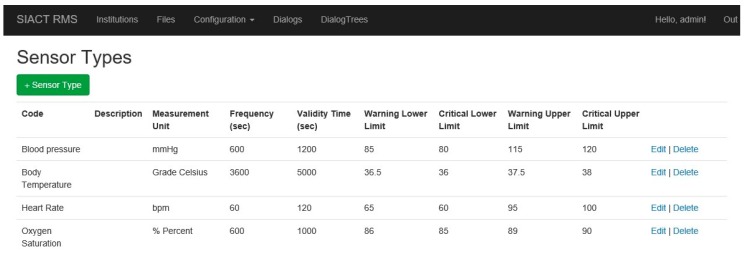
Defining the types of sensors used in the Patient WEB Application.

The list of sensors that are attached to the patient is editable at any time. Thus, it is possible to add new types of sensors for a particular patient. All sensors are defined in the “Configuration/Sensor Types” page of the application, illustrated in Figure 16.

**Figure 17:**
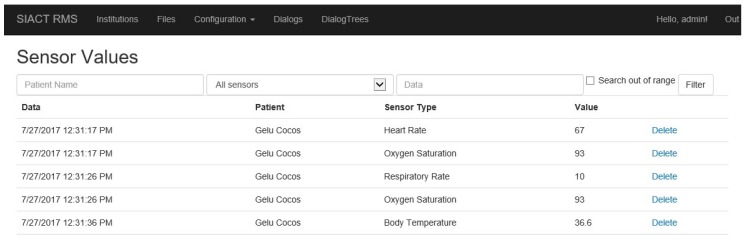
Data values provided by sensors mounted on the patient and recorded in the *Server* database.

The application displays a series of web services to which the Patient WEB Application can connect and transmit to the Server the values recorded by the sensors mounted on the patient. These values are loaded into the system asynchronously, so the services must be active 24 h a day. All of the data values received by the Server are viewed in the “Configuration/Sensor Values” page of the application, illustrated in Figure 17.

**Figure 18:**
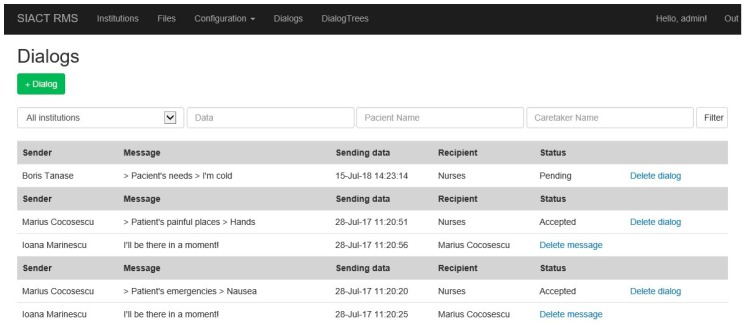
Dialogues between patients and caretakers recorded in the Server database.

The system administrator can view all dialogues between the patient and the caretakers using the “Dialogs” page in the application, illustrated in Figure 18.

Thus, the evolution of the patient’s condition and the medical and care services during hospitalization can be monitored. Messages cannot be erased from the database by supervisors (nurses, medical staff). Only the administrator has deleting rights.

#### 2.2.3. Software Component of *Caretaker Subsystem*

The Caretaker Subsystem consists of a Smartphone (or tablet PC), as illustrated in Figure 1, for communication with patients via the Server and real-time monitoring of the patient’s physiological parameters. The software application is written in Java for Android-enabled mobile devices using the JSON protocol, and it provides the following actions [40]:

- displays the keywords selected and transmitted by the patient in written or audio form;
- sends back the supervisor’s response to the patient;
- displays the values of the patient’s monitored physiological parameters;
- receives an alarm notification in audio and visual forms, triggered by the Server, when the normal values of the vital physiological parameters of the patients are exceeded;
- displays the time evolution of the physiological parameters’ values monitored during treatment.

The initiator of communication is always the Server Subsystem, which sends a notification when a patient has a need or a request. Every alarm notification includes the message ID, message priority, patient information, patient’s vital physiological parameters, patient’s message, and the caretaker/nurse’s confirmation once at the patient’s bedside [40]. The Server also sends notification alarms to the caretaker devices in case of emergency when normal values of vital physiological parameters are exceeded according to the alert detection algorithm implemented on the Server Subsystem.

The two-way communication protocols between Patient and Caretaker are as follows [40]:

(a) Normal operation when the caretaker reads and answers a notification alarm.
(b) Special situation (1) when the caretaker does not answer after reading the message.
(c) Special situation (2) when the caretaker does not read the notification alarm.

**Figure 19:**
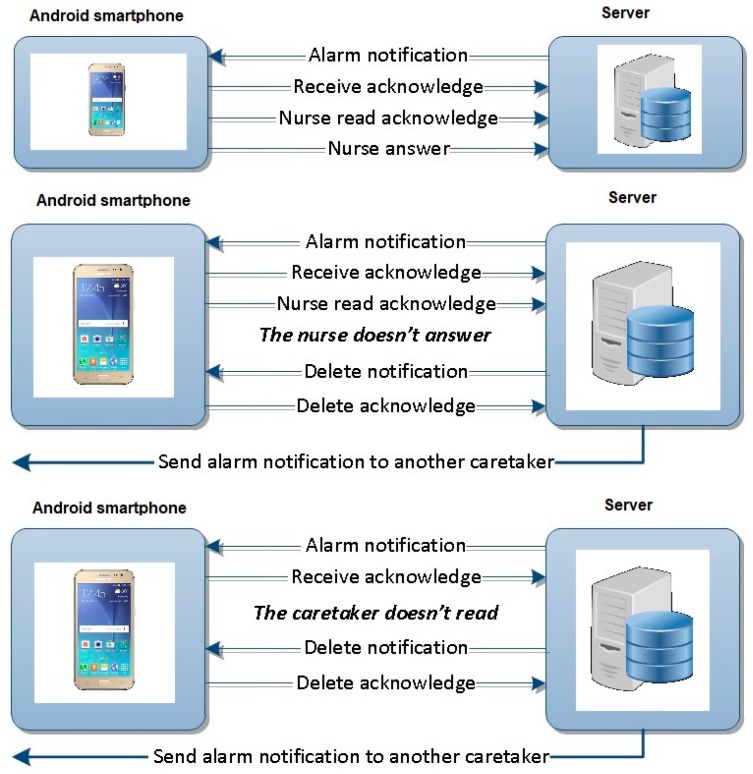
Two-way communication protocols between Caretaker device and Server Subsystem [40].

In cases (b) and (c), when the caretaker does not answer or read the notification within the scheduled time, the Server Subsystem will automatically send the message to another caretaker, deleting the one already sent to the first caretaker. This operating mode of the application ensures that an emergency expressed at any time is definitely resolved by a nurse. All communications are logged on the Server for further analysis. In Figure 19, the three communication protocols are illustrated [40].

One of the main advantages of this application is that one caretaker can assist several patients by communicating with them and telemonitoring their physiological parameters, and as a consequence, the cost of treatment can be reduced.

## 3. System Testing

The proposed assistive system was tested in laboratory conditions and in a hospital with different types of severely disabled patients.

### 3.1. System Testing in Laboratory Conditions

First, the proposed system was tested in laboratory conditions with healthy individuals for both functions of the system: communication and telemonitoring the physiological parameters.

#### 3.1.1. Testing the Communication Function

The communication function was tested for the detection of all types of patient needs using switch-type sensors and eye-tracking interfaces. All switch-type sensors and both types of eye-tracking interfaces illustrated in Figure 2 and Figure 3 were tested using PWA, which was personalized for detecting each type of patient’s needs, as shown in Section 2.2.1.

The communication function based on the switch-type sensor was tested very easily on all subjects, and it was learned in a very short time. During the tests, the frequency of the cyclical highlighting of the ideograms on the user screen was varied in order to determine the optimal value, i.e., appropriate for most subjects. Obviously, the optimal value of this parameter depends on the type of switch sensor used and can be personalized according to the user’s experience in using the system.

In order to implement the communication function based on the patient gaze detection, the following pupil detection algorithms were investigated and tested: LSFE, RANSAC, CHT, EHT, and PROJ (developed by the authors) and the open-source Starburst algorithm. These algorithms used by the eye-tracking interface were tested in different illumination conditions and for different operating scenarios specific to assistive technologies in order to implement the optimum software solution for the proposed system.

The accuracy of the analyzed pupil detection algorithms was tested on static eye images from different databases and on video eye images for real-time applications by using a new testing protocol developed for the scene image.

**Figure 20:**
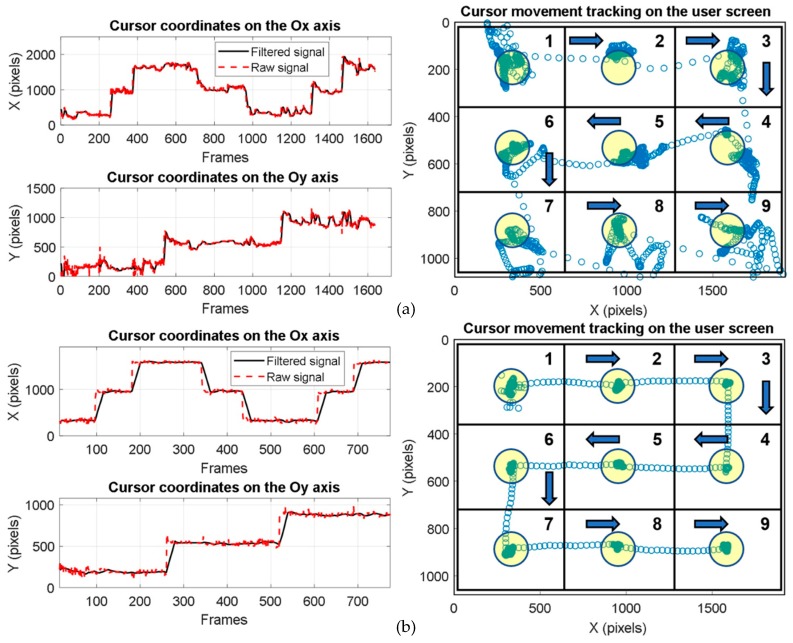
Signals provided by pupil detection algorithms (PDA) on both axes of the coordinate system and cursor movement tracking on the user screen: (**a**) non-experienced user; (**b**) experienced user.

The subjects who tested the system for a real-time scenario were required to keep their head in a fixed position and look at the user screen placed approximately 60 cm away. In this case, the test consisted of moving the cursor by gaze direction over nine identical quadrants in the order shown in Figure 20a,b while maintaining the cursor position as stable as possible in each circular target area with a 50-pixel radius (illustrated in yellow in Figure 20) located in the center of each quadrant; the cursor was to be maintained on the target for a certain period of time before moving to the next quadrant. The stationary time in each target area of each quadrant represents the dwell time used for simulating a mouse click. The performance of each algorithm was tested using the detection rate at 50 pixels in the scene (user screen) image. The detection rate at 50 pixels represents the number of frames (images) for which the Euclidean distance between the detected cursor position in a quadrant on the user screen and the center of the target circle placed in that quadrant is less than or equal to 50 pixels. According to the experimental results, the highest detection rate (91%) was obtained with the CHT algorithm. Considering the trade-off between accuracy, noise sensitivity, running time, and user ability, the optimum solution for real-time applications is the PDA based on the circular Hough transform implemented in our system.

The tests performed in laboratory conditions showed that subjects need significant learning time to gain the ability to use the eye-tracking system. It has also been found that there are people who do not have the necessary skills to use such a system, as shown in Reference [71].

Figure 20a,b illustrate the eye-tracking test results obtained with a non-experienced user and an experienced user, respectively.

Because of many noise sources (illustrated in Section 2.2.1) and the physiological eye movement during the eye tracking process, the vertical component of the signals provided by the PDA is more affected by noise than the horizontal one (Figure 20).

#### 3.1.2. Testing the Telemonitoring Function

The tests performed on the telemonitoring subsystem in laboratory conditions included two stages: assessing measurement accuracy and testing energy consumption.

**Figure 21:**
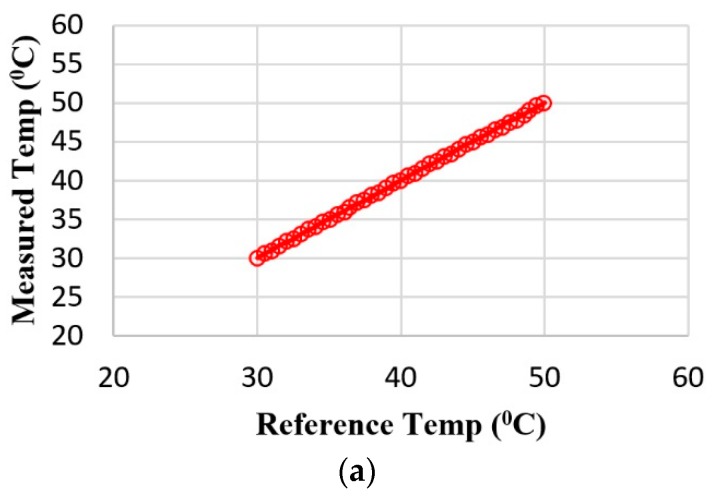

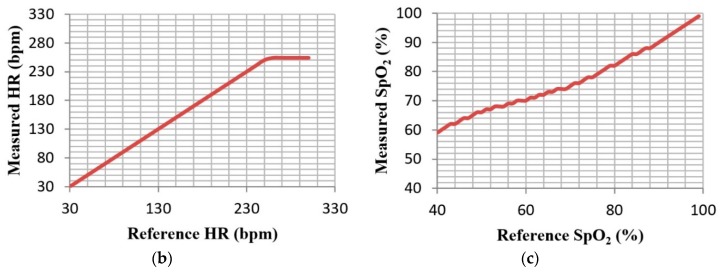
Testing telemonitoring function: (**a**) accuracy of body temperature measurements (°C); (**b**) accuracy of HR measurements (bpm); (**c**) accuracy of SpO_2_ measurements (%).

The accuracy of temperature measurement was assessed using a high-precision medical thermometer as a reference. The reference thermometer was placed in a thermally insulated enclosure with the body temperature monitoring device. The results obtained are presented graphically in Figure 21a. The data in Figure 21a show that the body temperature telemonitoring device measures the temperature with an error of less than ±0.15 °C, a tolerance suitable for medical measurements.

The accuracy of oxygen saturation (SpO_2_) and heart rate (HR) measurements was computed using the METRON SpO_2_ Analyzer simulator. The analyzer is usually used for the high-precision testing of commercially available pulse oximeters. The results obtained are illustrated in Figure 21b and show that the heart rate is calculated with the maximum possible accuracy in the range of 30–250 bpm. In Figure 21c, one can see that the SpO_2_ measurement error is less than or equal to ±2% for 80–99% SpO_2_, similar to that of a commercial pulse oximeter.

**Figure 22:**
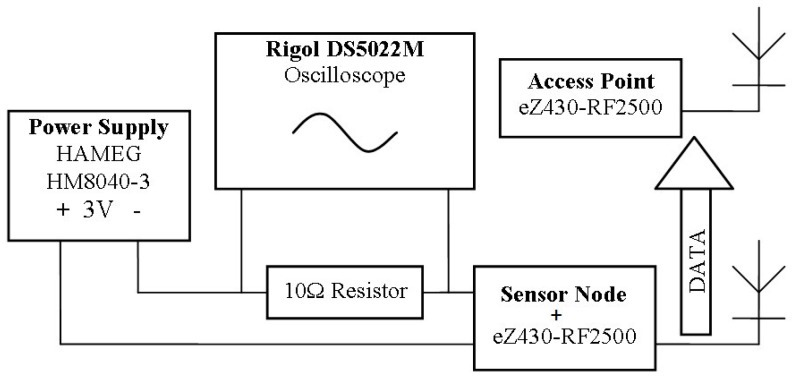
Evaluation of energy consumption.

For the evaluation of the energy consumption and battery life of each telemonitoring device, we used the configuration presented in Figure 22.

The power consumption of each telemonitoring device involves two components: power consumed by the acquisition (measurement) of signals (physiological parameters) and power consumed by the radio transmission component (eZ430-RF2500 wireless development kit). The transmission of the measured value to the Server Subsystem is performed at discrete time points (illustrated in Table 1) to optimize the power consumption of the radio module. After that, the device is switched to sleep mode. Also, the microcontroller of each sensor is switched to low-power operation mode at times of inactivity in order to preserve energy.

### 3.2. System Testing in Hospital

The end product is intended for use in hospitals (for acute cases), rehabilitation centers (for subacute and chronic cases), nursing homes (for elderly patients with severe handicap and multiple concomitant diseases), as well as the patient’s home (for chronic patients) in order to enable individuals with chronic conditions to remain independent and not require a nursing house.

The prototype of the proposed assistive system was tested in “Dr. C.I. Parhon” Clinical Hospital of Iaşi, Romania, on 27 patients hospitalized in the Clinic of Geriatrics and Gerontology in July 2017. The medical team involved in system testing comprised two doctors and two nurses.

The Ethics Board’s approval of our study is documented in the Agreement of the Ethics Board No. 4/12.07.2017 of “Dr. C.I. Parhon” Clinical Hospital of Iaşi, Romania.

The testing procedure consisted of the following steps:

1. Training the patient to use the system. Depending on the patient’s condition, the patient learning time varied between 10 and 20 min.
2. Testing both functions of the system: communication using keywords technology and real-time telemonitoring of the patient’s physiological parameters. Depending on the patient’s experience and cooperation, this stage varied between 20 and 30 min.

The system functionality tests were conducted independently for each patient, as the patients had different diseases, needs, capabilities, and learning times. One of the great advantages of the proposed system resides in its adaptability to very different patients’ needs. Depending on the degree of disability, patients can communicate using different switch-type sensors (illustrated in Figure 2) or eye-tracking interfaces (illustrated in Figure 3). In addition, the list of ideograms/keywords used by the Patient WEB Application can be configurated according to the patient’s needs and level of understanding. Also, the physiological parameters telemonitored can be customized according to each patient’s needs.

#### 3.2.1. Participants

The mean age of the patients included in this study was 72.27 ± 8.23 years; the minimum age was 55 years old and the maximum age was 89 years old. The proportion of female patients was 76.9%. The frequently associated pathologies were degenerative osteoarticular diseases (73.1%) and cardiovascular diseases (34.6%). Some patients had more than one condition leading to disability. All patients were able to complete a questionnaire regarding the usage of the new system. The relevant demographic details are described in Table 2.

The inclusion criteria were the following: (1) male and female hospitalized adults; (2) the presence of documented comorbidities leading to disability, such as neurodegenerative disorders, chronic severe diseases that are associated with a high degree of handicap (cardiovascular disorders: congestive heart failure, severe valvular disease, severe arterial disease, chronic pulmonary diseases, previous stroke, or other neurological diseases associated with disability, amputations, etc.); (3) preserved ability to answer questions regarding the system usage; (4) a cooperative attitude and ability to use the system.

The exclusion criteria were the following: (1) refusal to sign the informed consent form; (2) the presence of a significant visual disorder; (3) the presence of documented severe neurocognitive impairment; (4) the presence of unstable major medical conditions; (5) the use of medication that could interfere with the patient’s ability to test the system.

All patients included in the study population underwent a general medical evaluation, and comorbidities were found in previous medical records or the patients’ charts. The general medical evaluation was performed by the geriatric medical team that participated in the study. All of the patients read and signed the informed consent form before any measurements were taken. The study team informed the patients about the testing procedures and all activities, and they agreed to perform them for the purpose of testing the system.

#### 3.2.2. Experimental Sessions

In order to check all functions of the proposed assistive system, three types of tests were carried out on the patients, as shown by the testing plan detailed in Table 3.

The communication function based on a switch-type sensor was tested with all six types of switch sensors listed in Table 3. The sensor type was chosen according to the degree of disability of the patients who tested the system. This mode of patients’ needs detection is used for disabled patients who can perform some controlled muscle contractions, such as moving a finger, forearm, or foot; inspiration/expiration; sip/puff (patients with partial paralysis, paraplegia). The test consisted of sending several messages (composed by the testing team and including keywords from different database hierarchy levels) to the supervisor using the switch-based communication mode of PWA. The task was considered to be carried out when the patient’s request was received by the caretaker device and the supervisor sent a response that was displayed on the patient’s screen. This procedure required a minimum learning time (10 min). All patients with discernment were able to use this type of communication.

The communication function based on eye tracking interface has been tested for both types of eye tracking interfaces illustrated in Table 3. This type of patient’s needs detection is used for severely disabled patients who cannot perform any controlled muscle contraction, apart from the eyeball movement and blinking (complete paralysis, tetraplegia, myopathies, amyotrophic lateral sclerosis, etc.). The test consisted of sending several messages (composed by the testing team) to the supervisor using the eye-tracking-based communication mode of the PWA. The task was considered to be carried out when the patient’s request was received by the caretaker device and the supervisor sent a response that was displayed on the patient’s screen. This procedure required a medium (15 min) or long (20 min) learning time depending on the type of eye-tracking interface used. Only patients with ocular disorders (strabismus) failed to successfully use the eye-tracking interface.

The telemonitoring function was tested by all patients. The test consisted of using a wireless network of medical devices (wearable sensors) mounted on the patient’s body for the acquisition of the following physiological parameters: blood oxygen saturation, heart rate, respiratory rate, and body temperature. Data values provided by the sensors are transmitted by the patient’s device to the Server, where they are recorded and can be viewed with the PWA (Figure 17). As needed, the system can also monitor other physiological parameters, such as blood pressure (BP) and galvanic skin reaction (GSR).

The hospital testing of the telemonitoring function consisted of tracking the acquisition and correct transmission of the measured value to the Server database and from the Server to the caretaker’s mobile device. The values recorded in the database (Figure 17) were compared with those registered by the hospital equipment, and a 98% similarity was obtained.

The alerting of the supervisor in special situations when the normal values of the monitored parameters were exceeded was also tested using the alert detection algorithm. This procedure does not require any patient training, so the learning time is not applicable.

Regardless of the test procedure stage, a test was immediately stopped if requested by a patient, a member of the medical staff, or a legal guardian. All actions undertaken by the team performing system testing were done only with the patient’s consent and for their benefit. During the tests, the confidentiality of the patient’s identity was respected by anonymizing personal data.

## 4. Results and Discussion

To assess the functionality of the proposed system, the patients who participated in testing answered a questionnaire that focused on its necessity, usefulness, operating modes, flexibility, and adaptability to the patient’s needs.

The questionnaire prepared by the testing team included the following questions for patients:

1. Do you consider that the system responds to your needs for communication with the supervisor/others?
2. How do you evaluate the operating modes of the system?
3. How do you evaluate the two functions of the system (communication and telemonitoring of physiological parameters)?
4. Do you think that the keywords that run on the screen respond to your needs?
5. How do you evaluate the graphics used by the system?
6. Do you consider the hierarchically organized database useful?
7. How do you rate the selection of keyword/ideogram by using the eye-tracking technique?
8. How do you rate the selection of keywords/ideograms with the switch-type sensor?
9. Do you think the ideograms that accompany the keywords are suggestive?
10. Do you consider that you were able to communicate effectively with the supervisor during the tests?
11. Do you consider the telemonitoring of the physiological parameters useful?
12. Do you think the system is flexible enough to quickly reconfigure to your needs (the frequency of highlighting the ideograms/keywords, modifying dwell time, changing databases to enter or remove keywords, changing icons or sounds that accompany keyword displaying)?
13. Do you consider the system tested to be useful in the hospital/at home?
14. What was the level of fatigue experienced during testing?
15. Did you feel physical discomfort due to the different modules of the system you tested?

**Figure 23:**
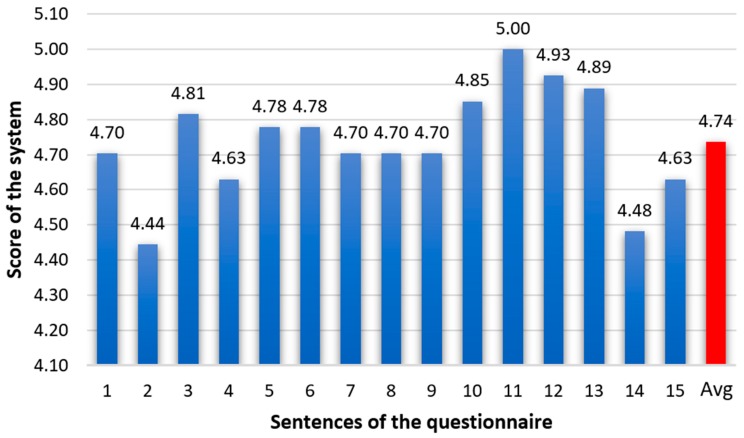
Testing scores obtained by the proposed system at “Dr. C.I. Parhon” Clinical Hospital of Iaşi, Romania.

For questions 1–13, the patients’ response was scored on a scale between 0 (strongly disagree) to 5 (strongly agree). The answers to questions 14 and 15 were measured on a scale between 0 (the highest level of fatigue/discomfort) and 5 (no fatigue/discomfort). The value corresponding to the patient’s evaluation was scored in the response table. The average scores of the patients’ responses to each question are illustrated in Figure 23.

**Figure 24:**
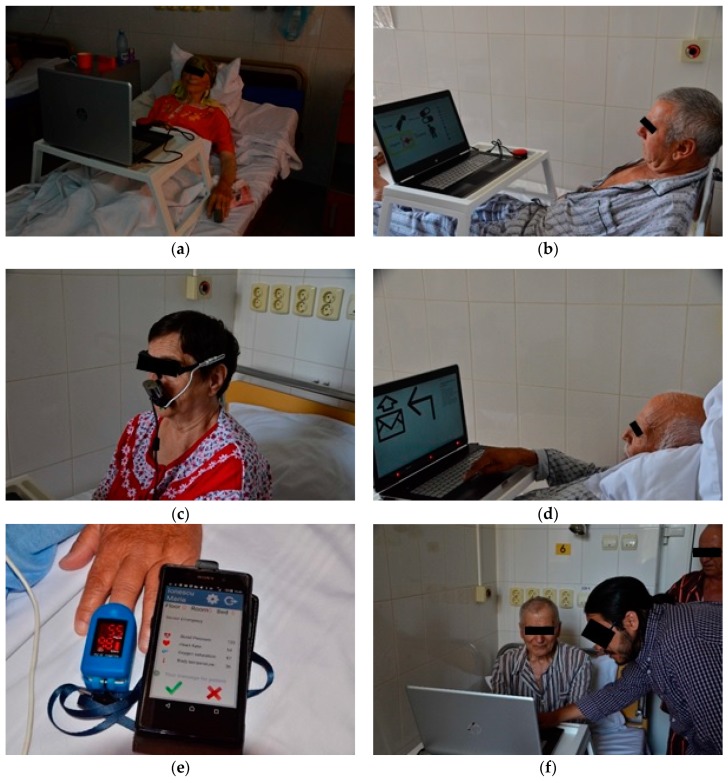
System testing on hospitalized patients at “Dr. C.I. Parhon” Clinical Hospital of Iaşi, Romania. Testing the communication function based on a switch-type sensor (**a**), (**b**); using the head-mounted eye-tracking interface (**c**); using the remote eye-tracking interface (**d**); testing the telemonitoring function by the measurement of blood oxygen saturation and heart rate values and the transmission of these values to the caretaker device; (**e**) training the patients to use the system (**f**).

Figure 24a–f are images taken during the testing of the system with hospitalized patients at “Dr. C.I. Parhon” Clinical Hospital of Iaşi, Romania.

The communication rate depends on the type of communication used by the system (using the switch-type sensor or the eye-tracking interface) and the patient’s physical condition. It varies greatly from patient to patient and also within the patient’s training period.

For the communication function based on the switch-type sensor, the communication rate depends on the cyclical highlighting speed of the ideogram, with the speed varying between 1 ideogram/s and 1 ideogram/10 s according to the patient’s ability. On the other hand, the duration of the selection depends on the ideogram position in the scene image and in the keywords database hierarchy. As a result, for a keywords database with several levels of hierarchy, the duration of ideogram selection can vary from a range of 1–4 s to a range of 80–120 s. Of the total number of patients, 15 participants opted for a highlighting speed of 1 ideogram/3 s. They succeeded in making the selection in the first scroll cycle. Of the patients who failed to make the selection on the first attempt, nine patients opted for a highlighting speed of 1 ideogram/5 s and three patients selected 1 ideogram/10 s. Only one participant (3.7% of the total) was unable to complete the task because of difficulties in understanding.

For the communication function based on the eye-tracking interface, the communication rate depends on the dwell time. According to Päivi Majaranta, expert eye typists require only a very short dwell time (e.g., 300 ms), while novices may prefer a longer dwell time (e.g., 1000 ms) to give them more time to think, react, and cancel the action [72]. Experimental results have shown that disabled patients also prefer to use a long dwell time [73]. For this reason, in our tests, the dwell time varied between 1 and 3 s depending on the user’s ability to use the system. This range of values was experimentally determined, according to the needs of patients who tested the system, and is in line with the results reported in References [73] and [74]. In Reference [73], it is shown that the dwell time threshold for people with disabilities depends on patient’s physical condition; the same dwell time may be “short” for one user and “long” for another. Thus, the system must be flexible in order to accommodate a higher range of dwell time values and different needs of the users with various disabilities. Of the total number of patients, three participants (11.1% of the total) were unable to complete the calibration stage successfully and, as a consequence, they could not test this function of the system. Two other participants (7.4% of the total) could not use the head-mounted eye-tracking interface because it was too difficult to keep their head in a fixed position. Of the patients who tested the communication function based on the eye-tracking interface, three participants opted for a dwell time of one second, five opted for two seconds, and 16 opted for three seconds. Compared with other commercial systems, ours has the advantage of being able to change this time according to the user’s experience in using the system. On the other hand, patients with stroke and neurological diseases had difficulty in using the eye-tracking interface.

Furthermore, the communication rate through eye tracking also depends on the running time of the pupil detection algorithm used in the system. To be suitable for real-time applications, the algorithm must have a speed that is higher than 10 frames/s. The PDA based on the circular Hough transform used in our system provides a speed of 20 frames/s; thus, it is a user-friendly algorithm and suitable for real-time applications.

The system usability was assessed by the testing score obtained by the system (presented in Figure 23) and by the task success rate (Table 4).

In the evaluation of system performance, 10 participants gave the maximum score (5), and the lowest result was 3.67. Thus, the overall mean score was 4.74, with a standard deviation of 0.38.

For the communication function, the task success rate represents the percentage of the patients who successfully accomplished their task.

For the telemonitoring function, the task success rate represents the ability of the system to correctly transmit the measured values to the Server database and from the Server to the mobile device of the caretaker. The transmitted values are compared with those registered with the hospital equipment, and the obtained similarity (expressed as a percentage) represents the task success rate of the telemonitoring function.

## 5. Conclusions

The assistive system proposed in this paper was developed as a functional and testable prototype that enables two-way communication with severely disabled patients while it simultaneously telemonitors the patients’ vital physiologic parameters.

The proposed assistive solution contributes to the development of techniques and technologies for monitoring and communicating with severely disabled patients. It provides the means of a new communication method for people lacking normal abilities, to whom it offers adequate procedures based on keywords technology and equipment. The system also contributes to the development of techniques and technologies for telemonitoring the vital physiologic parameters of disabled patients.

Furthermore, the proposed assistive system contributes to the acquisition of new knowledge about disabled people’s contact behavior and communication with intelligent machines by using a computer–human interface—a difficult field to study with a clear interdisciplinary nature—and a combination of proper ergonomic design, communication techniques and technologies, and postoperative care aspects.

The proposed assistive system presents a series of apparent advantages compared with other similar ones: (1) the innovative principles of two-way communication between severely disabled patients and caretakers/medical staff by gaze detection techniques based on video-oculography; (2) the possibility of investigating patients with severe disabilities who cannot communicate with other people by means of classical communication (spoken, written, or by signs) by using the communication technique that makes use of keywords/ideograms organized in several hierarchical levels; (3) the ability to implement advanced communication by alphanumeric characters, thus allowing severely disabled patients to use the Internet and e-mail; (4) the ability to dynamically adapt both the software and hardware structure according to the patient’s needs, the evolution of their condition, and medical recommendations; (5) the permanent monitoring of several physiological parameters, their analysis, and the alerting of caretakers in emergency situations, all of which are carried out using the same resources as those used for communication; (6) the increased accountability of medical personnel as a result of logging caretaker interventions; (7) lower caretaking costs due to fewer caretakers needed to assist a larger number of patients.

Many attempts and tests (both in the laboratory and on patients) were performed in order to obtain an optimal solution for the prototype. The final tests of the prototype were performed at “Dr. C.I. Parhon” Clinical Hospital of Iaşi, Romania, with patients from the Geriatrics Clinic.

Although the number of patients included in prototype testing was small, we consider them highly representative of the population of Romanian seniors with severe disabilities and low quality of life.

The test results demonstrate the system’s good performance in both of its primary functions: communication with disabled patients and telemonitoring their physiological parameters. Both the medical staff and patients involved in system testing positively evaluated its functions, ease of use, and adaptability of the system to the needs of patients, highlighting its utility in a modern medical system.

The main benefits of our assistive system that are conferred to disabled people include the patients’ improved health and wellbeing, their faster reinsertion into society, an increase in the quality of medical services, a decrease in patients’ expenses, and an increase in the number of medical services afforded by ambulatory medical care.

The results obtained make us confident enough to continue this study in other medical facilities that care for people with severe disabilities, particularly patients with neuromotor disabilities.

## Figures and Tables

**Table 1:** Data transmission rate, energy consumption, and battery life.

Telemonitoring Device	Data Transmission Rate	Average Current Consumption	Battery Capacity	Days of Operation
HR and SpO_2_	1 measurement/10 s	6.1 mA	1250 mAh	8.50
Respiratory rate	1 measurement/10 s	0.14 mA	240 mAh	71.0
Body temperature	1 measurement/10 s	0.20 mA	240 mAh	45.5

**Table 2:** The demographic details of the study population.

	Included (*n* = 27)
Age	72.27 ± 8.23
Female	20 (76.9%)
Cardiovascular diseases	9 (34.6%)
Pulmonary diseases	1 (3.8%)
Stroke	3 (11.5%)
Other neurological diseases	2 (7.7%)
Degenerative osteoarticular diseases	19 (73.1%)
Amputations	1 (3.8%)

**Table 3:** Testing plan: tested hardware/software components of Patient Subsystem, patients’ diseases, and learning time depending on system function.

Tested System Function	Tested Hardware Components of the Patient Subsystem	Tested Software Components of the Patient Subsystem	Patients’ Diseases	Patients’ Learning Time
Communication	Switch-type sensor: - hand switch-click; - pal pad switch; - ribbon switch; - wobble switch; - foot switch; - sip/puff breeze switch.	- PWA (keywords technology for switch-type sensor)	Disabled patients who can perform some controlled muscle contractions, such as movement of a finger, forearm, or foot; inspiration /expiration; sip/puff, etc. (partial paralysis, paraplegia, amputations, congestive heart failure, degenerative osteoarticular diseases, stroke, chronic respiratory failure, severe COPD, recovery after major surgery, etc.)	Minimum (10 min)
	Eye-tracking interface: - head-mounted device; - remote device.	- PWA (keywords technology for eye-tracking interface); - eye-tracking algorithm (including virtual keyboard and Internet/e-mail browser).	Severely disabled patients who cannot perform any controlled muscle contractions apart from eyeball movement and blinking (complete paralysis, tetraplegia, myopathies, amyotrophic lateral sclerosis, stroke, severe degenerative osteoarticular disease, severe arthritis in the shoulder, amputations, etc.)	Medium or long (15–20 min)
Telemonitoring	Wireless body area network: - oxygen saturation; - heart rate; - respiratory rate; - body temperature.	- *SimpliciIT* protocol; - GUI for telemonitoring the physiological parameters.	All patients	-

**Table 4:** Task success rate.

	Communication Function Based on Switch-Type Sensor	Communication Function Based on Eye-Tracking Interface		Telemonitoring Function
		Head-Mounted	Remote Device	
**Task success rate**	96.3%	81.5%	88.9%	98%
